# 236. Effects Of a Blood Culture Diversion Device on Blood Culture Contamination Rates and Clinical Outcomes - A Multi-Center Study

**DOI:** 10.1093/ofid/ofad500.309

**Published:** 2023-11-27

**Authors:** Anthony J Febres-Aldana, Patrycja Ashley, Eva Amenta, Megan M Duffey, Theresa Sepulveda, Theresa Sepulveda, Sabra L Shay, Miriam Barrett, Todd M Lasco, Takei Pipkins, Bradley Lembcke, Margaret Reed, Mayar Al Mohajer

**Affiliations:** MD Anderson Cancer Center, Wesley Chapel, FL; Baylor College of Medicine, Houston, Texas; Baylor College of Medicine, Houston, Texas; Baylor College of Medicine, Houston, Texas; Baylor College of Medicine, Houston, Texas; Baylor College of Medicine, Houston, Texas; Premier Inc, Colorado Springs, Colorado; Baylor St Luke's Medical Center, Houston, Texas; Baylor St. Luke's Medical Center, Houston, Texas; Baylor St Luke's Medical Center, Houston, Texas; Baylor St Luke's Medical Center, Houston, Texas; Baylor St. Luke's Medical Center, Houston, Texas; Baylor College of Medicine, Houston, Texas

## Abstract

**Background:**

The use of initial specimen diversion devices (ISDD) has been shown to reduce blood culture (BCx) contamination. Studies have estimated that each false-positive BCx costs $3,073-4,818, based on an increased length of stay (LOS) of 1-8.4 days and antimicrobial and laboratory use; however, it is unknown whether implementing ISDD would lead to a reduction in these outcomes. This study aimed to assess the impact of ISDD utilization on BCx contamination, LOS, antimicrobials days of therapy (DOT), and false positive central-line associated bloodstream infections (CLABSI) due to Coagulase-negative Staphylococcus, enterococci, and Candida.

**Methods:**

This retrospective study included patients at three medical centers. In center 1 (treatment arm), BCx were initially collected without ISDD (pre-intervention). The first Plan-Do-Study-ACT (PDSA) cycle included utilizing an ISDD (Steripath®) for BCx collection; however, the uptake was low, given the absence of process metrics. In the second PDSA, BCx was only accepted by the laboratory if submitted along with the ISDD wrap. In centers 2-3 (control), BCx were collected without ISDD across the three periods. Difference-in-difference (DID) was used to estimate the effect of PDSA2 on outcomes by comparing the changes between the treated and untreated groups (Figure 1). BCx contamination, CLABSI, LOS, and DOT were entered into multiple regression models to adjust for demographics and several comorbidities.

**Results:**

A total of 15,810 patients were included. Groups had similar characteristics across the study periods except for SARS-COV2 and admission status (Table 1). After adjusting for confounders (Table 2), the intervention was associated with a reduction in BCx contamination (OR 0.38, P< 0.001), no change in LOS (B=0.08, P=0.620), and increased DOT per patient days (IRR=1.10, P=0.006). There were 8 CLABSIs in the treated group (2 preintervention, 3 PDSA1, and 3 PDSA2) vs. 1 in control (PDSA2), and the model did not converge due to the small number.

**Figure 1:**
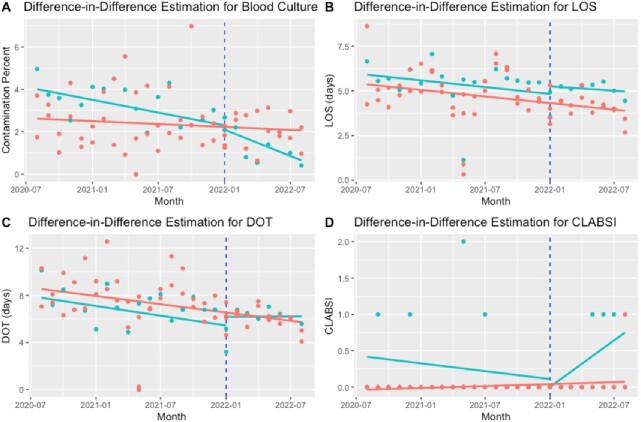
Difference-in-difference estimation for blood culture contamination (A), LOS (B), DOT (C), CLABSI (D) LOS: Length of Stay; DOT: Days of Therapy; CLABSI: Central-line associated bloodstream infections The blue vertical line represents the start of the second Plan-Do-Study-ACT (PDSA2) The green and red points represent monthly data per treatment and control groups, respectively. The green and blue lines represent the average per each group before and after PDSA2.

**Table 1:**
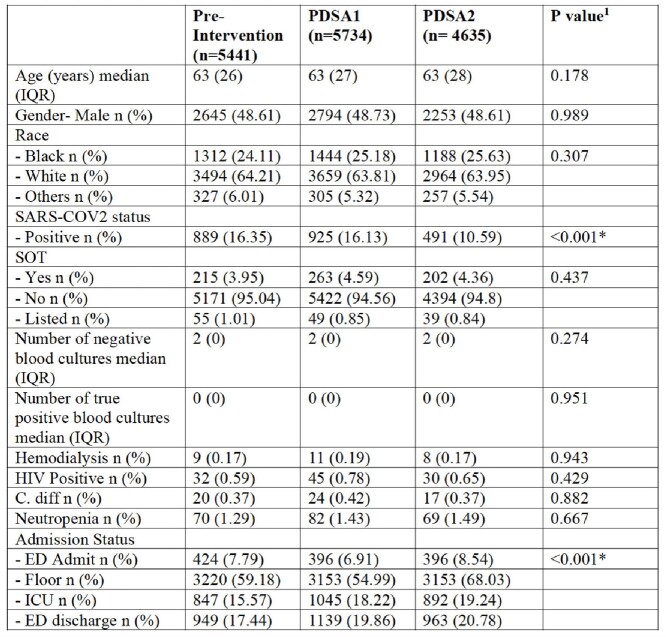
Patient Characteristics Across the Study Groups. PDSA: Plan-Do-Study-Act; IQR: Interquartile Range; SARS-COV2: Severe Acute Respiratory Syndrome Coronavirus 2; SOT: Solid Organ Transplantation; HIV: Human Immunodeficiency Virus; C. diff: Clostridioides difficile; ICU: Intensive Care Unit; ED: Emergency Department 1 Kruskal-Wallis or Chi-squared tests were used for statistical comparison as appropriate. * Indicates statistical significance (P<0.05).

**Table 2:**
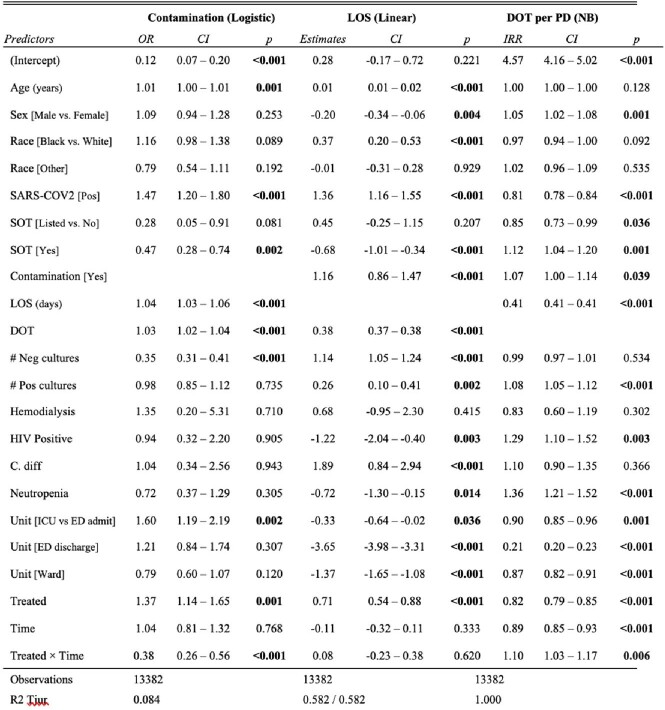
Multiple Variable Regression for Blood Culture Contamination, Length of Stay and Days of Therapy. NB: Negative Binomial; OR: Odd Ratio; CI: 95% Confidence Interval; LOS: Length of Stay; DOT: Days of Therapy; PD: Patient Days; IRR: Incidence Rate Ratio; Pos: Positive; Neg: Negative; SARS-COV2: Severe Acute Respiratory Syndrome Coronavirus 2; SOT: Solid Organ Transplantation; HIV: Human Immunodeficiency Virus; C. diff: Clostridioides difficile; ICU: Intensive Care Unit; ED: Emergency Department.

**Conclusion:**

Implementing ISDD decreased BCx contamination without reducing LOS, DOT, and CLABSI. Further studies are needed to assess if implementing ISDD would lead to clinical benefits and establish the true cost-effectiveness of this method.

**Disclosures:**

**Sabra L. Shay, BSN, MPH**, Premier Inc.: Employee

